# Virtuous Journalism

**Published:** 1888-01-21

**Authors:** 


### VIRTUOUS JOURNALISM.

By an Old Matron.

I have been much interested in some articles in the *Lancet* on medical ethics in relation to popular journalism to which my husband has drawn my attention. He is not a medical man, but, like many others, he reads the *Lancet* at his club. The articles, I am sure, are in reality directed against The Hospital. It is true that your writers are attacked personally, though not by name, rather than your journal, on the pretence that it is contrary to good ethics for medical men to write articles in semi-popular journals. It is more than hinted that such articles are really advertisements, and are not inspired, as they profess to be, by a desire to make the lay public familiar with the first principles of health and disease.

I see that the colleges are taking the matter up, and are about to call their graduates to account for these contributions. It is not for me to criticise this determination, but I must say that I do not see the consistency of either the lament of the *Lancet* or of the colleges. On what grounds will the contributors to a book called "The Book of Health" (which we bought in consequence of what the reviewer of the *Lancet* said of the book) escape condemnation? The opening chapter is written by the President of the College of Surgeons, and the next article by the former President of the College of Physicians. Will these writers get off by reason of their high position? If not, who will be the spokesman of the colleges, seeing that their highest officials are themselves partakers in undertakings which, at the instigation of the *Lancet* (of all journals!), they now so roundly deplore and condemn? Will they argue, because "The Book of Health" costs a guinea and The Hospital one penny a-week, that there is any real difference in the aims of the two? Does not the editor of the former say in his preface that the book is intended for "the general public." Surely this is not another attempt to set the classes against the masses! In my ignorance I should have thought that it is the masses who mostly require the help which your paper affords, for it is amongst them that the greatest ignorance prevails; and are they to be deprived of this help because they can only afford to take in their information in a weekly form instead of *en bloc* in the guise of a guinea book! When any of my children get low or look weakly I feel awfully ignorant. Do you think that the *Lancet* takes this matter so much to heart because it has always had such a large circulation outside doctors, and fears that The Hospital and the other journals may interfere with this? My husband tells me that it is more difficult to get to see the *Lancet* at his club on Saturday afternoon than the *Saturday Review*, such a favourite is it with many gentlemen beside himself.

But the *Lancet* will surely see that to be consistent they must now put a stop to their lay circulation, and carefully restrict their sales to medical men, otherwise their contributors will bring themselves into contact with the college edicts and authorities. The *Lancet* is to be seen at every bookstall, with a staring bill of contents! This is not the only difficulty. The articles in the *Lancet* are not (professedly) written for the lay public, although I am told there is much difference of opinion even among medical men on this point. They are likely, therefore, if read—and they are largely read—to do mischief. But articles in The Hospital, or in "our contemporary" (I have seen many instructive ones in the latter, which have formed subjects for articles for the *Lancet* subsequently, on account of their general interest I presume) and elsewhere, when written purposely for us lay readers, are capable of affording a great amount of most useful information.

I am quite at a loss to see the harm, or that it is at all derogatory to the dignity of any man, however high, to write articles and sign them in journals such as this, provided they are adapted to the understanding and capacity of the average reader. There is no way likely to reach the public most interested in these questions, at once so short and so direct as by means of a well-conducted journal, and I think no way so likely to put a stop to the unhealthy craving to read special journals like the *Lancet* now largely prevalent. The *Lancet* is a strictly-professional journal, and contains articles only suited for professional readers. Why do the proprietors—medical men—cater so much for the general public? Do you ever see the society journals? Have you not read about a certain kind of "soap which won't wash clothes"? You will recognise it by the presentment of a monkey in full dress, admiring himself in a polished frying-pan, across which may be read, "See what the *Lancet* says!" Is not the moral obvious?

